# Book Reviews

**Published:** 1825-09

**Authors:** 


### CRITICAL ANALYSIS

OF

### ENGLISH AND FOREIGN LITERATURE,

RELATIVE TO THE VARIOUS BRANCHES OF

### Medical Science.

Quæ laudanda forent, et quæ culpanda, vicissim

Illa, prius, cretâ; mox hæc, carbone, *notamus*.—PERSIUS.

### DIVISION I.

### ENGLISH.

ART. I.—*An Essay on Tetanus, founded on Cases and Experiments.* By JOSEPH SWAN, Member of the Royal College of Surgeons, and Surgeon to the Lincoln County Hospital.—8vo. pp. 98. Longman and Co. London, 1825.

A WORK on Tetanus cannot fail of exciting attention. The pathology of the disease is so little understood,—its result is so generally fatal,—the sufferings of the patient during its continuance are so severe,—and the train of symptoms so formidable to behold, that it must necessarily at all times arrest the attention of the medical inquirer; whilst his interest and his humanity equally demand his most strenuous endeavours to penetrate the mystery in which it is enveloped, and to establish, if possible, some rationally-grounded method of cure. That the present work of Mr. SWAN can be said to have accomplished either of these much-to-be-desired objects, we are reluctantly compelled to deny, but the attempt at least is laudable; and we are induced to hope that, having once begun this inquiry, our author will, upon reflection, see how little he has really accomplished, and be stimulated to further exertions. His zeal in the pursuit of his profession is unquestioned, his abilities we much respect, but, in our humble opinion, he has hitherto been too prone to publish without fully digesting his materials, or reflecting whether the inferences he has deduced are fairly the result of the facts he has so laudably and industriously collected. To him we should be inclined to apply (with the change of one word) the advice which the younger PLINY gives to his correspondent—"Multum *scribendum esse* non multa." That this censure is not unjust, it will now be our business to show, by presenting our readers with a detailed account of the contents of this volume; for, although seated in the critical chair, we by no means consider our opinions as infallible, and still less do we wish that our readers should take any thing for granted upon our simple assertion.

To begin, then, at the beginning. From the second paragraph in our author's Preface, we are led to think that he ascribes too much importance to anatomical investigations; for he says, "This imperfect knowledge (that is, of the nature of tetanus,) is, I think, to be ascribed to the insufficient minuteness in our examinations of bodies after death." (P. 4.) In fact, the whole novelty of the present work consists in the establishment of an anatomical fact relative to the appearances on the opening of the bodies of three persons who died in consequence of tetanus; the first of which examinations occurred to our author in May 1823, who perceived in that case an unhealthy appearance of the ganglia of the grand sympathetic nerve. In November, his second dissection of a tetanic subject took place, and the same appearances presented themselves: this was again the case in examining the body of a patient of Mr. THOMAS MACAULAY; and we are told that M. ANDRAL, fils, also observed great redness in the semilunar ganglion in a patient who died of fever, with symptoms of tetanus.

After alluding to the written opinions of Dr. GOOD and Mr. ABERNETHY, as relating to this view of the subject, our author continues thus:

"If what I have written amounts to the same as is expressed in the preceding paragraph; if, likewise, some of the opinions I have advanced are the same as those advanced by Dr. Good, and there is any merit in the following pages, I shall willingly rest satisfied in having proved, by dissection and experiment, what those gentlemen have so ingeniously proposed.

"It is a matter of considerable moment to ascertain how the body is usually affected by injuries and diseases; and, although I cannot presume on doing this in a way by any means conclusive, yet, as it is impossible to understand the nature of tetanus without a certain degree of knowledge respecting the changes produced in the body by injuries and diseases, I thought it necessary to premise my observations by an inquiry into the nature of constitutional irritation." (P. 9.)

Having thus stated the outline of his work, Mr. Swan proceeds, in the first chapter, to treat on *constitutional irritation*. The object of this chapter is to show that, where severe injuries have been received, the whole body suffers in consequence; and that there is not only a disordered state of the functions of important parts at a distance from the seat of the injury, but that a great change in their appearance is discovered on dissection. To illustrate this undeniable position, two cases are related; the first of a child, seven years of age, who was burnt on the thighs, arms, and back, by her clothes taking fire on the 20th August, 1823: the skin of the abdomen was not implicated in the accident. The next day, there was pain in the belly and vomiting; the intellects were not much impaired, but the face was deathlike; the pulse not to be felt, and she was cold. Purging medicines were given, but they were not retained on the stomach. In the evening, the pulse was perceptible, but she was insensible; the pupils of the eyes were dilated, and did not contract on the approach of light. Six leeches were applied to the temples. She died at one o'clock in the morning of the 22d.

It is not our intention to make many remarks on the practice adopted in this case, because our objects are totally distinct; but we may be permitted to say that, with a cold skin, no pulse, and a death-like countenance, purgatives do not seem to have been indicated: nor does the re-action in the evening, when the pulse was perceptible, seem to have been such as to have demanded the application of leeches. We say this merely *en passant.* We are not informed whether this case occurred in our author's practice or not; and it is most likely that, under any circumstances, a fatal event could not have been averted. But to return to the business immediately before us.

The examination of the body showed the lungs very purple, and loaded with blood; there were spots of ecchymosis behind the posterior mediastinum on the left side; there was a great vascularity on the outside of the aorta, and some fluid in the pericardium. In the abdomen, the only appearances of unequivocal disease was, that about six inches of the jejunum were in a high state of inflammation.

"All the ganglia of the grand sympathetic nerves in the chest were vascular. On the right side, the semilunar ganglion, and all the rest of the ganglia in the abdomen, were very vascular. On the left side, the semilunar ganglion, and the first in the abdomen formed by the continuation of the grand sympathetic nerve, were very vascular, but the others were not. The nerves of each axillary plexus were very vascular. The sciatic nerves within the pelvis, and the anterior crural nerves, were vascular, but not near so much so as the axillary plexus.

"After every accident in which the constitution sympathises with the injured part, I believe the ganglia of the grand sympathetic nerves become irritated, and the functions of the parts supplied by them with nerves are disturbed in consequence. The action of the heart is increased in proportion to this degree of irritation in them, so long as it continues moderate." (P. 14.)

The second case is that of an old lady, upwards of eighty, who fell down and fractured the neck of the thigh-bone, on the 14th of November, and who gradually sunk under the effects of constitutional irritation; the leading symptoms being pain in the hip, thirst, costiveness, difficulty in making water, and want of sleep. Latterly she complained of great tenderness in the abdomen, and died insensible on the 19th of December. The body was opened at seven in the evening.—We pass by our author's description of the fracture of the thigh-bone, and proceed to the following passage:—

"There was violent inflammation of the semilunar ganglia. The viscera of the chest were sound. The outside of the aorta was very vascular. All the intestines were very vascular, as if a state of excitement bordering on inflammation had existed in them. The bladder was very large, and contained some urine. The uterus was very hard, and had a small ossific tumor in the fundus. The labia were excoriated; and there was a similar appearance on the buttock of one side." (P. 17.)

Next in order we arrive at the detail of several experiments upon animals, made with a view of observing the effects of constitutional irritation upon internal parts. These experiments are nine in number, and the subjects of them were large healthy dogs;—they consisted in the insertion of certain pieces of arsenic or gamboge (the weights of these pieces being specified,) into wounds made in the back, between the shoulders of these animals; or in inflicting compound fractures upon their limbs. We need not go through the detail of these experiments seriatim. The main facts are the following:—Arsenic, when inserted into a wound, appears to have occasioned involuntary twitchings of the muscles, and locally to have excited much inflammation round the point of insertion. Both the first dogs were hanged,—the first, five days from the commencement of the experiment, the second at the end of two days. In both, the appearances on dissection were similar, differing only in intensity. The ganglia of the grand sympathetic nerve were inflamed; the par vagum had a greater redness than usual; the thoracic viscera were sound; but the stomach and intestines were in a state of inflammation in the one case, and of ulceration in the other.

Four other experiments were then made with gamboge, instead of arsenic, the effects of which were to cause a purging and to excite thirst; and the degree of these effects seemed to be regulated by the size of the piece of gamboge employed. In the third experiment, fifty-four grains and a half were used, and the animal appeared on the fifth day to be recovering; whereas, in the fifth experiment, seventy-six grains were inserted, and the dog died, after a violent spasm of all the muscles of the body, on the following day, or about twenty-five hours after the insertion of the gamboge. It is to be observed, that the three last animals were shot, instead of being hanged; our author observing that hanging produces a greater vascularity, resembling inflammation, in the internal parts, if life is not almost immediately extinguished.

With respect to the appearances found upon examining the bodies of these animals, it will be sufficient to remark, that, in all, vascularity of the ganglia of the sympathetic nerve, with inflammation of the stomach or some portion of the intestinal tube, in a greater or less degree of violence, was invariably observed; neither the brain nor medulla spinalis affording any remarkable diseased appearances.

Of the three dogs whose legs were fractured, the first was hanged after two days, the second after three, and the third at the termination of ten days. In all the same condition of the nervous ganglia were observed, excepting in the last instance, where the semilunar ganglion only was affected.

These experiments are interspersed with the detail of two cases; one of which is related, in order to prove that, when a serious accident has happened, and particularly if any of the vital organs have been injured, there is frequently no re-action in the system, especially if venesection has been much employed; and we then do not find any alteration in the appearance of the nerves. Yet how does this proposition agree with the first case contained in this volume, in which, after an extensive burn, death ensues in less than forty-eight hours after the accident, and certainly without any thing like re-action; and yet here we have a general vascularity of all the ganglia of the grand sympathetic nerve. Such is the consequence of attempting to draw general conclusions from isolated facts.

The other case is ushered in by the following remark, and, as it is short, we give it insertion:—

"In consumptions and complaints attended by hectic fever, I have not found an increased vascularity of the ganglia of the grand sympathetic nerves; but, if any thing happens to bring on acute inflammation, the symptoms change, and may cause an increased action in the ganglia, as in the following case.

"Case.—Robert Norris, æt. ten years, began to be unwell at Midsummer 1822, and kept gradually declining. For some time before he died, his pulse was 120. He had a purging, but no cough, and his tongue was red. He made much urine. He ate voraciously, and always vomited after. He had profuse perspiration. He complained of much pain in his head, which began ten days before he died. For the last three or four days he was quite insensible. He died on the 25th of April, 1823." (P. 44, 45.)

The appearances on dissection are related at length; but it is sufficient for us to remark, that the ganglia of the sympathetic nerve in the chest were very vascular, as was the semilunar and the other ganglia in the abdomen, though in a less degree.

We had almost forgot to mention that the last experiment recorded, that of inserting a flat piece of oxide of arsenic, weighing thirty-nine grains, into a wound on the left side of the middle of the back of a puppy, was followed, on the fourth day, by a paralytic condition of the hind legs. The animal was drowned the evening of that day; and, on examining the body, inflammation was found to extend some distance round the wound, but did not pass the middle of the back. The arsenic had only lost half a grain in weight. But here, though there was much fluid contained in the chest, and the pleura of the left lung was highly inflamed and covered with coagulable lymph, the ganglia of the grand sympathetic nerves were not in the least inflamed. The brain and medulla spinalis were natural.

Our author, in order to obviate any objection that may be raised as to these appearances of the nervous ganglia being really produced by various medicines and diseases, observes, that he has examined an executed subject immediately after it was cut down, and found the ganglia of a pearly appearance, and free from any mark of vessels carrying red blood. He has found one very red, and the corresponding one very white, and this in so marked a degree as to leave no more doubt of its being the effect of inflammation, than the inflamed appearance of the conjunctiva of one eye would do when contrasted with the natural appearance of the other.

In concluding this chapter, our author asks how can an almost similar appearance of increased vascularity in these nerves be caused by different medicines and diseases, and produce such various symptoms? He does not answer this question.

The second chapter of our author's work treats of *Idiopathic Tetanus*, and, after a few preliminary remarks on the division of this disease by nosologists, we come to the relation of a very interesting case of idiopathic tetanus, for which Mr. Swan is indebted to his friend, Mr. T. Macaulay.

The patient was a boy, ten years of age, who first perceived a stiffness in his neck on the morning of the 30th of November, 1824: he had previously enjoyed good health. He experienced no pain, and it did not appear to attract much attention. In the course of the day he was exposed to a very bleak air, and, shortly after his return home, he was suddenly seized with a violent tetanic spasm of the greater part of the body. No assistance was sought until the following day, though in the interval the spasm never relaxed, and he did not get any sleep in the night. On the 1st of December, he was found by his medical attendant lying upon the bed in a state of perfect rigidity: the opisthotonos was complete; he had a little power over the motions of the left leg, but not of the right; the same was observable with regard to the arms. Touching any part of the body would frequently produce a spasm; blowing on the face, or attempting to swallow fluids, produced most violent spasms of the throat, neck, and jaw. The jaw was not permanently closed, but any attempt to protrude the tongue produced a most violent spasm; the face was flushed, and the countenance anxious and wild; the pulse ninety and sharp, but the intellects were not affected. He complained only of pain in the back, at the commencement of the dorsal vertebræ; pressure increased it, and seemed also to accelerate the spasms. He was costive; the tongue was white, and has had much thirst. He was bled from the arm to the amount of twelve ounces, by which he was relieved in all respects; twelve leeches were applied to the back, and a purgative of calomel and jalap given every two hours. At half-past nine, the countenance was altered for the worse, though the lad thought himself better: the spasm, however, continued, and the jaw was more affected than in the morning. No effect from the powders; the blood drawn was not inflamed,—the coagulum, indeed, was remarkably loose and thin; the pulse 150, but not sinking; no power whatever over the right leg or arm. He was quite sensible. Six grains of calomel were administered, which produced vomiting, and it was repeated with one of the powders ordered in the morning; twenty-four leeches were applied to the back, and a purging glyster every two hours. About one o'clock, before the leeches were applied, and whilst in the act of receiving an enema, the spasms relaxed, and he expired.

The body was examined eleven hours after death, and a considerable volvulus was found in two portions of the intestines. The villous coat of the small intestines throughout had marks of having been in a state of great irritation: it was loaded with green and yellow slime; and, at the upper extremity of each volvulus, several lumbrici were lodged, and others were found in different parts of the canal. The ganglia of the great sympathetic nerve were minutely examined: in all these were decided marks of irritation,—they were very vascular. The left semilunar ganglion exhibited a few vessels; but the right was injected in a beautifully minute manner. In the head, every part of the pia mater was found minutely injected with blood; there was some fluid in the ventricles and at the base: on removing the brain, about two ounces were found; and, on hanging the head over the edge of the table, the fluid kept dribbling from the spinal canal, the medulla of which was quite healthy. The sheath of the dura mater seemed to be distended with fluid, but, on being divided, was found to contain only air. The limbs of the right side remained quite stiff; those of the left were in a considerable degree relaxed.

Such are the most essential particulars of this very interesting case, which we have been obliged to abridge, leaving out, we believe, however, no point of great importance. It is certainly one of the strongest and best-marked cases of tetanus, arising from the presence of worms, we ever met with.

The second case we shall merely notice, by saying that Mr. Swan doubts whether the disease was tetanus; and the quick, and we are afraid we must also add, the successful termination, inclines us very much to be of his opinion.

Another case is next recorded by our author, taken from Lobstein's recent publication, and which is a true specimen of the disease, caused by cold taken in the rainy season of October; but what is most remarkable is, that, in the examination of the body, a very distinct inflammation of the semilunar ganglion is mentioned to have been observed. The same fact is also recorded by M. Andral, fils, in two individuals, who died of what the French term ataxo-adynamic fever, and one of whom presented, within the last forty-eight hours of his life, violent trismus, and a tetanic rigidity of the superior extremities.

This chapter concludes with an account of three experiments made upon dogs, with the alcoholic extract of nux vomica; the main facts contained in which we shall briefly mention. The dogs experimented upon were of different sizes, and consequently the effect of the medicine was different, both in degree as well as in the period of time requisite for the destruction of the animal. Tetanic spasms were produced in all. The first animal, a small bitch, required four grains, given at three separate times, to kill her. The second dog, of a large size, resisted the effects of repeated doses, (each of which, however, produced convulsions,) from the 21st of October to the 1st of November; in which time she had swallowed forty-three grains, the first dose being one grain, the last six grains. The third animal was of a moderate size, and lived under the exhibition of various doses, from one to four grains and a half, from the 22d of November to the 6th of December. In all, the morbid appearances were the same essentially: redness and vascularity of the ganglia of the great sympathetic nerve, and the same appearance in some portions of the par vagum. There was also, in all three instances, increased vascularity of the vesssels of the pia mater.

*Traumatic Tetanus* forms the subject of the third chapter of Mr. Swan's work, which commences thus:—

"If traumatic tetanus always followed a very painful or extensive wound, there would be an apparently satisfactory reason for its violent symptoms; but, as it likewise supervenes on a trifling injury, or a wound that is nearly or entirely healed, there is the greatest difficulty in comprehending how it is produced.

"With a view of removing, as much as possible, this obscurity in the production of traumatic tetanus, I have been induced to inquire how the body is usually affected after accidents. From that inquiry I have been led to state that, when a severe injury has been received, the ganglia of the grand sympathetic nerves become irritated, and consequently the parts to which they distribute nerves. When the constitution is healthy, I believe the irritation of the ganglia goes off in a few days, and then the parts supplied by them with nerves return to a state of quietude, and again perform their healthy functions.

"When the ganglia of the grand sympathetic nerves have been thus affected, and the irritation has subsided, an unhealthy action in the wound may excite a fresh irritation in them. Or, even if the wound be healed, the passions, improper food, and other causes, may continue, reproduce, or increase, the disordered state of the organs receiving nerves from the ganglia, and thereby excite a fresh irritation in them." (P. 75, 76.)

Upon this passage we shall only observe, that the facts recorded in the previous chapters do not at all bear out our author in these assertions. It is true, indeed, that he has shown the ganglia of the great sympathetic nerve to be irritated or inflamed in consequence of severe injuries; but that is the whole amount of what he has shown. He has failed to prove the other parts of his proposition. An extensive burn kills an infant,—the nervous ganglia are found inflamed, but there is no appearance of tetanus. An old woman dies from fever after a fall,—the ganglia are inflamed, but there are neither spasms, convulsions, nor tetanus; neither can we suppose that in her case the irritation would have gone off from goodness of constitution; and therefore she ought to have had tetanus, for the parts supplied by the ganglia with nerves could not have returned to a state of quietude again. In the animals experimented upon, arsenic, gamboge, nux vomica, all produce the usual effects of inflammation upon the internal organs and upon the ganglia of the nerves; and yet, with the exception of those in which the nux vomica was employed as a poison, no tetanus was produced.

If we quit speculation, and look at practical facts, we shall find that by far the most numerous instances of recovery from this disease are wholly irreconcileable to the belief that its essence consists in inflammation. What do the records of medicine prove on this subject?—that ammonia, wine, brandy, opium, &c. have all been employed in large, and sometimes even in excessive doses; and these are precisely the cases which have most commonly been followed by recovery. If we are called upon for our authorities, we refer the reader to Dr. Good's work, to the Dictionnaire des Sciences Medicales, to the Transactions of various learned Societies, and to most of the periodicals of modern date. It is undoubtedly true that bleeding to a very great extent has occasionally been resorted to,—tobacco injections have also been recommended,—mercury, the warm and the cold bath, have each had their advocates; and that here and there a recovery has followed the use of all or each of these means; but still the principle upon which the majority of cures has been performed, is a tonic and stimulating plan of treatment. So that we cannot doubt, even from the evidence given by the author himself, that tetanus does not solely consist in an inflamed condition of the ganglia of the sympathetic nerve or par vagum; but that there is still a mysterious something superadded, which remains unexplained, and which will produce this terrible disease after a slight and apparently trifling injury; when, in many cases where we should naturally expect it to appear, no such occurrence takes place. This we have witnessed more than once: nay, often it will suddenly come on when a wound is not only healthy and healing, but actually healed, and without any of those marks of stomach derangement, or, in fact, without affording us any clue by which to account for its presence; for the pain at the stomach, which almost always precedes the attack of traumatic tetanus, is in fact the precursory symptom, and truly a link in the chain of diseased actions.

We make this last remark a little prematurely, since it arises out of the relation of a case of traumatic tetanus, detailed by our author, in which a boy, aged twelve, was severely burnt by the explosion of some fire-works in his pocket, on the 5th of November, 1823, and who went on well till the 14th of the month, when the sore was beginning to heal. On the 13th, he had no appetite; on the next day, he complained of pain in the stomach; and, on the 15th, the jaw became fixed. Death took place on the following day.

From this case our author concludes, that, as on the 13th a want of appetite was complained of, the digestive organs had been by some means disordered. We think quite differently. If any slight disorder of the digestive organs is to be the exciting cause of tetanus after injuries or accidents, who would escape? We believe the failing in the appetite, and the pain at the stomach, to be really the commencement of the tetanic attack; and we again repeat that, in those cases of traumatic tetanus that we have seen, this distressing pain about the epigastrium has never been absent.

We shall not detain our readers with an account of the appearances on opening the body of this young gentleman, as they do not differ from what has often been stated before: neither shall we extract the case immediately following, which has already been published in another work* by the same author: it is one of those instances in which amputation was resorted to without effect. The morbid appearances are such as

* *On the Action of Mercury on the Living Body.*

have been mentioned in the preceding pages, though in a more violent degree.

We have now gone through the whole of our author's cases and experiments, and have only to consider the observations which he makes upon some of the symptoms of tetanus,—the parts that appear to be implicated in the disease,—and then to inquire what inferences may be drawn from them, as leading to the most probable means of cure. These considerations occupy the last nine pages of the volume. Mr. Swan first remarks, that the causes of the spasms, or at least of their continuance, is not to be found in the injured part; that the muscles implicated are supplied by nerves from the brain and medulla spinalis; and yet the functions of the former of these organs are seldom disturbed, sometimes not even to the last. Noticing the increased vascularity of the pia mater, our author is led to believe this to be only an effect; and he argues that this could not be inflammation, or we should frequently see its consequences: nor would so complete a recovery take place, either from the disease in the human subject, or from leaving off the extract of nux vomica, when violent tetanic spasms had been produced by it, in the animals experimented on. Now here we must observe, that, although these arguments equally apply to the vascularity of the ganglia of the nerves, as to that of the pia mater, it may only be an effect. There is equal absence of all the usual results of inflammation; and, as to the argument derived from the exhibition of nux vomica, is it not gratuitous to call such spasms tetanic? "Every like is not the same," is very sound philosophy, though written by a poet. These reasons apply to three or four subsequent pages of our author's remarks; but we give the following paragraph in his own words:—

"According to the violence or mildness of the disease, I conceive the irritation is confined to the ganglia and some of the cerebral and spinal nerves for a certain space; it is then communicated to the membranes of the brain and medulla spinalis, and sometimes causes so great an effusion of fluid as must add to the danger, and may produce sudden death.

"No very important anatomical facts relating to this subject have been recorded by medical authors; there are few complaints, therefore, in which the method of treatment has been more empirical, and few in which the termination has been more generally fatal.

"The facts I have adduced are both curious and important, and therefore are, perhaps, entitled to some notice; but I cannot presume on their being sufficient of themselves to enable us to form any decided rules of practice, and it is therefore with much reluctance I offer any observations on this part of the subject.

"From the appearances on dissection of patients who have died of this complaint, I cannot help concluding that there is a state of parts bordering on inflammation, and therefore that general blood-letting is indicated. Fever, and other decidedly inflammatory symptoms, may not generally be present, yet they sometimes exist in a very great degree, as was most particularly exemplified in an interesting case* related by Mr. Earle." (P. 92—94.)

The functions of the digestive organs are also to be attended to: an emetic is probably indicated, and afterwards the patient should be purged. We must recollect that some high authorities are directly adverse to strong purging in this disease. With regard to mercury, our author doubts its propriety; as, in experiments upon animals, he found it produced inflammation of the nervous ganglia, such as he observed in tetanus; and we must say that he is borne out in this opinion by the little benefit that has been practically gained by the employment of that remedy. He mentions the probable good effects of the compound powder of ipecacuanha, not recollecting, perhaps, that Dr. LATHAM has strongly advocated its use. Finally, he suggests that the meadow-saffron might probably have some influence in the cure of the disease.

In concluding our notice of Mr. Swan's work, we have only to say, that the announcement of the peculiar appearances he has described in the ganglia of the great sympathetic nerves and the par vagum, is the only point of interest which it contains; and we must recollect that his evidence only goes to confirm what had been previously observed and stated by LOBSTEIN, though this theory had long since been started by FRANK, and has had numerous advocates both in this country and on the continent.

---

ART. II.—*Remarks on Irritative Fever, commonly called the Plymouth Dock-yard Disease; with* Mr. DRYDEN's *detailed Account of the fatal Cases, including that of the lamented Surgeon,* Dr. BELL. *Dedicated, with permission, to* Commissioner SHIELD. By JOHN BUTTER, M.D. F.R.S. F.L.S. and W.S. Fellow of the Royal College of Physicians in Edinburgh, and of the Royal College of Surgeons in London; Member of many Medical and Phrenological Societies, in London, Edinburgh, Paris, Philadelphia, &c. &c.; President of the Western Medical and Chirurgical Society; and Physician to the Plymouth Eye Infirmary.—8vo. pp. 302. Underwoods, London, 1825.

OUR readers may, perhaps, require to be told that the fever which is the subject of the work now before us, excited much interest, and no little alarm, in Plymouth and its neighbourhood, in the autumn of last year. The alarm has now subsided; but, to the medical inquirer, a relation of the principal circumstances

* *Medico-Chirurgical Transactions,* vol. vi. p. 93.

attending that fatal disease cannot fail of proving highly acceptable, since it involves many questions in pathology. The disease seems to have borne some relation to those cases of irritative fever produced by slight wounds in dissection and from other causes, which have lately attracted so much attention, and received great illustration from the labours of Dr. Colles, Dr. Duncan, jun. and Mr. Shaw, as well as from the publication of many isolated, but extremely valuable, facts, by Mr. A. T. Thomson, Mr. Anderson, Mr. Travers, Dr. Nelson, and others.

Dr. Butter's motive in presenting this work to his professional brethren, is excellent. Unconnected with the medical establishment of the dock-yard, the labourers of which have been the subjects of this unfortunate visitation, he has been induced, with the assistance of Mr. Dryden, surgeon to the dock-yard, to collect the cases, and to accompany them with his own comments, together with all the concurrent circumstances that could tend to throw any light upon the subject, in order to elicit from his medical friends a more full discussion; and his object has already been answered, in as much as it has enabled us to present to our readers, in this very Number of our Journal, the detail of two cases at full length, of one of which our author was not able to do more than offer a sketch. That the writer of those cases should have drawn conclusions different from those to which Dr. Butter has arrived, is not matter of surprise to us, and seems to have been anticipated by the author himself, from one or two expressions which we find in his Preface.

We have already stated the motives which induced our author to step forward as the historian of this fever. The cases related, we are informed, were drawn up by Mr. Dryden; and, in order to render them as correct and full as possible, the widows and friends of the deceased artificers were waited upon by the writer and Mr. Dryden, in order that the histories might be made as accurate as possible by the most frequent revision and care. Yet it is to observed, that these cases were not originally noted down with any view to publication; though our author believes that, though some minutiæ might have been kept back by the parties concerned, the general outline of the facts cannot be mutilated by trifling omissions. (P. xi.) Some delay, it appears, occurred in the publication from the want of type, it being the first work of the kind ever printed in the town of Devonport; and we have pleasure in saying, that the execution of it is very creditable to the persons employed.

With respect to the arrangement of the work, we are inclined to think that it would have been rendered more useful had it been less bulky. That portion which relates to the supposed causes of irritative fever, is greatly too much extended; much of the matter contained in it bears little or no relation to the subject in hand; and much is transcribed from recent publications, to which a reference would have been sufficient. Under the head of *general treatment,* we conceive that it would have been better to have simply related what was done in the cases before us; the reasons that prompted the treatment, and remarks on its result; rather than the lengthened disquisition on the general effects of certain remedies in other instances of irritative fever, which might or might not bear some relation to the dock-yard disease. These general objections we urge to the form of the work, without, however, meaning to quarrel with our author for adopting a different mode: he has followed the plan which appeared to him to be best; and, if we find fault, it is only, as we have before said, because we should have preferred a plain statement of the cases and the mode of treatment, with such remarks as arose incidentally from those histories, to the more diffuse and complex form which Dr. Butter has given to his labours.

Leaving this unprofitable discussion, however, we proceed to examine the volume; and, in order that our readers may the better understand the subject, we shall proceed at once to page 122, where we are informed that the number of mechanics employed in the dock-yard at present is about two thousand, most of whom, from their employments, are much exposed to accidents; their hours of labour are from six in the morning to the same hour in the evening in the summer, and from day-light until dark in the winter; they take their meals at eight, twelve, and nine o'clock, and live temperately. When ill of fever or otherwise, they receive no pay; and, when injured by hurts contracted at their work, half-pay; so that they have no inducement to report themselves sick. In the course of each year, it is calculated that between three and four thousand men hurt themselves, so as to apply for surgical relief; but of that number only about four hundred are incapacitated from attending their duty. It appears that, during ten years preceding 1824, only two instances had occurred of men dying from fever supervening on local injuries. All these preliminary matters it is necessary to relate, in order to a right understanding of what follows; and we think this explanation should have preceded the detail of the cases.

It appears that between the 24th of June and the 31st of December, 1824, above 250 men were laid up from their duty, by reason of various hurts received at work; several of whom had inflammatory attacks of no great consequence, but only fifteen were affected with disease in its malignant form, of whom twelve died. In addition to these fifteen cases, it appears that Dr. Bell, the surgeon of the dock-yard, fell a victim to a fever of the same character, which arose from a wound which he received in opening the body of George Nicholls, one of the sufferers. Mr. Dryden also wounded himself upon more than one occasion, after opening the bodies of the men who died, and suffered from irritation of the skin over his hands; so that, in the course of one night, blisters would arise, containing a bloody or grumous sanies, which, being discharged, would be followed by a thickening and livid colouring of the surrounding skin: finally, he suffered much from a carbuncle, which began over the carpal extremity of the radius.

Our author presents us with evidence, in the shape of a letter from Dr. Cowan, of the Portsmouth dock-yard, to prove that, though erysipelatous inflammation is a very frequent sequel of local injuries received in dock-yards, fever, gangrene, or death, are rare occurrences; not one death having been caused by those diseases, in consequence of hurts or other injuries, in the space of twelve years at Portsmouth.

We now go back to the cases themselves, first presenting our readers with a synoptical view, *(for which see opposite page,)* which was drawn up by Mr. Blewitt, a pupil of Mr. Dryden's, which shows the periods of invasion of the disease, its connexion with the local injury, and its termination.

It is quite obvious that we cannot afford space for the detail of many of these cases; but the relation of those of Quick and Walkie in the former part of this Number, would render it almost unnecessary for us to do so, since the case of Walkie is, perhaps, one of the strongest marked in the whole catalogue. We shall, however, relate two more: that of William Butters, because it is short, has a decided connexion with the previous accident, and the subject of it appears to have enjoyed uniform good health previously, to have been in all respects a temperate man, and it is one of the three instances of recovery. This we give as a contrast to that of John Bate, who, in point of constitution, was just the reverse of the former.

"Case III.—John Bate, æt. forty-five, shipwright, working as a labourer, sallow complexion, strumous appearance; residing in Princess-street, Devonport.

"On the 16th of August, 1824, had the point of his left fore-finger jammed off by a plank, and immediately amputated at the last joint. Cathartic pills given.

"17th.—Perfectly easy. Cathartic mixture ordered.

"18th and 19th.—Doing well.

"20th.—Complains of pain in the hand, and of head-ache; pulse about ninety-six, and full. Dressings removed, and cataplasms substituted. Twenty-four ounces of blood were taken from his arm.

"21st.—The stump easy, and looking inactive; pulse 110, weak;

*Synoptical View of the alarming Cases which occurred in his Majesty's Dock-yard at Devonport, during the year* 1824.

| *No.* | *Names.* | *Age.* | *Local Injury.* | *When received.* | *Commencement of Fever.* | *Its Duration.* | *Result.* | *When died, or returned to Duty.* |
|---|---|---|---|---|---|---|---|---|
| 1 | William Cowle | 45 | Puncture from a nail in his left foot | Between 25th and 31st July | 1st August .... | About 15 days | Death .... | 15th Aug. |
| 2 | John Henwood | 45 | Laceration of his hand by a saw.... | 4th August.............. | 6th August .... | 67 or 68 days .. | Do. ...... | 11th Oct. |
| 3 | John Bate .... | 45 | Left fore-finger jammed off by wood | 16th August ............ | 20th August .. | 5 days ........ | Do. ...... | 25th Aug. |
| 4 | William Butters | 40 | Nail torn off his right ring-finger .. | 10th August ............ | 18th August .. | About 4 weeks | Recovery | 11th Oct. |
| 5 | Robert Horne | 32 | Left great-toe bruised............ | 30th August ............ | 1st September | 15 days ...... | Death .... | 14th Sept. |
| 6 | Gregory Nichols | 45 | Abrasions on his right shin-bone .. | 7th, 8th, or 9th September | 13th September | 6 days ........ | Do. ...... | 19th Sept. |
| 7 | William Lobb | 40 | Chin contused by a nail .......... | 13th September .......... | 18th September | Nearly 2 months | Recovery | 29th Nov. |
| 8 | John Rawling.. | 40 | Three fingers of right hand jammed | 17th September .... ..... | 19th September | 6 days ........ | Death .... | 25th Sept. |
| 9 | Dr. Bell ...... | 58 | Scratch on his right fore-finger .... | 19th September.......... | 20th September | 5 days ........ | Do. ...... | 24th Sept. |
| 10 | John Walkie .. | 38 | Abrasion on his inner ankle ...... | 13th September.......... | 1st October .. | 5 days ........ | Do. ...... | 5th Oct. |
| 11 | William Reeves | 44 | Abrasion on his left shin-bone .... | 9th October ............ | 3d November .. | About 12 days | Recovery | 27th Dec. |
| 12 | William Taylor | 42 | Laceration of right great-toe by a saw | 2d August .............. | 9th August .... | 5 days ........ | Death .... | 14th Aug. |
| 13 | John Quick .. | 37 | Fracture of his right great-toe .... | 18th August ............ | 22d or 23d Sept. | 6 days ........ | Do. ...... | 29th Sept. |
| 14 | John Long .... | 37 | Contused finger and abraded leg .. | 27th August ............ | 8th September | 2 days ........ | Do. ...... | 10th Sept. |
| 15 | Thomas Beer .. | 40 | Hand wounded by glass .......... | 14th September.......... | 19th September | 5 days ........ | Do. ...... | 24th Sept. |

tongue white and moist. Calomel and opium given with confection at bed-time, and cathartic mixture in the morning.

"22d and 23d.—Boluses continued.

"24th.—Great prostration of strength, anxiety, and nausea. A blister to the epigastrium; cathartic mixture repeated.

"Five o'clock P.M.—Symptoms much aggravated; the pulse scarcely perceptible at the wrist; breathing oppressed; abdomen tense and painful to the touch; singultus. Castor-oil and tincture of senna given immediately, and repeated after two hours. Fomentations to the abdomen, and enemata, ordered.

"Nine o'clock P.M.—Profuse perspiration; breathing excessively oppressed: he is evidently sinking.

"25th.—Died at nine o'clock A.M.; rational almost to the last.

"*Dissection.*—Body examined nine hours after death.—The thorax contained about sixteen ounces of serum. The right lobe of the lungs tinged with blood, and bound with strong adhesions to the pleura costalis. The left lobe black, disorganised, easily torn, and apparently gangrenous. The pericardium rather vascular, containing about two ounces of fluid; the heart sound.

"The intestines were in general excessively distended with gas, particularly the colon and cœcum; no fæces; the whole highly inflamed, particularly the lower portion of the ilium and cœcum, which in some places approached to gangrene. On removing the intestines, a tumor was perceived on the right side, which contained nearly a pint of pus, and was found to surround the right kidney, that viscus being apparently healthy; the left kidney also sound. The liver was much enlarged, very soft, turgid, easily lacerated, and with difficulty separated from the side. The gall-bladder distended with bile. The spleen and pancreas healthy. The stomach also healthy, except a red spot, about an inch in diameter, on the posterior or vertebral side, near the pyloric orifice.

"CASE IV.—William Butters, æt. forty, a joiner, and a very healthy man, possessing an excellent constitution, who has been always most temperate in his living; residing in Chapel-street, Stonehouse, with his wife and six children.

"On Tuesday, the 10th of August, 1824, whilst planing a piece of mahogany in the joiners' shop of his Majesty's dock-yard at Devonport, he tore up the nail of his right ring-finger by a splinter of wood. Dr. Bell removed the nail on his application at the surgery, and ordered poultices, which were continued to the finger for five days, and afterwards light dressings for three days; he, Butters, continuing at his work during that time.

"This accident happened the more easily in consequence of the infirm state of the nails of the ring and middle fingers of the right hand, owing to a fracture of the last phalanges, produced by their being jammed between two pieces of timber in February last. He was then confined six weeks by these fractures, but suffered no unusual constitutional irritation.

"On Wednesday morning, 18th of August, whilst dressing himself, he felt shivered and unwell, but nevertheless went to the dock-yard:

whilst there he became so ill with general disorder, rigors, and faintness, that he was obliged soon to quit the yard, previous to the usual hour of departure. He applied to Dr. Bell, who remarked that his illness could not depend on the finger, which on this day looked healthy and granulating. Poultices were again applied to the finger, and cathartic medicines ordered.

" 19th.—His bowels had been freely opened. He was somewhat easier.

" 20th.—His fever had increased, with severe head-ache. His hand and arm were now much inflamed; the sore felt cold and smarting, rendering the connexion, which had been before doubtful, between the local injury and the constitutional disturbance, both manifest and certain. VS. ad ℥xxxiv. Medicam. Cathartic. cum Ant. tart. subinde. Fotus partibus affectis.

" 21st.—The blood, drawn yesterday by Mr. Dryden, buffed; fever not diminished; head-ache unsubdued. His head was now so disordered, that a sort of redness *(photopsia)* prevailed before his eyes, and a phantom as though a piece of wood was encircled by fire,—signs highly indicative of great cerebral excitement. Hand and arm very painful, much swollen, and erysipelatous. The redness appeared in patches and lines on the inner side of the arm in the course of the absorbent vessels, which were red even to the axilla. Great œdema over the whole arm. Admoveantur hirudines xvi. temporibus. Repr. mistura cathartica. Sumat nocte bol. sequent.

℞. Pulv. Antimon. gr. vj.
Ext. Opii gr. ij.
Confect. q. s. M.

" 22d.—The leeches afforded relief to his head. Symptoms as yesterday. Continuerentur cataplasmata et fotus.

" 23d and 24th.—No particular alteration.

" 25th.—The whole hand and arm still erysipelatous, and greatly swollen in an increased degree, pitting like dough on pressure; his pulse rapid and weak; strength greatly exhausted; head-ache rather lessened, but still very bad; tongue moist and clean. On the back of his right hand an obscure fluctuation was now perceptible, which Mr. Dryden opened by an incision nearly two inches in length; the cellular tissue was injected with pus. Habeat ol. ricini ℥j. statim.

" 26th.—The tension of the whole arm was lessened, and the head-ache relieved, after the opening; the redness disappearing.

" 27th.—Improving. Ol. ricini ℥j. statim.

" 28th, 29th, and 30th.—Better in every respect. Repr. oleum; habeat mistur. cathartic. pro re natâ.

" September 2d.—Soft dressings and light bandages were re-applied to the finger and arm, in lieu of the poultices, &c. which had been continued up to this period over the whole limb.

" 8th.—Convalescent. Owing, however, to excessive debility, and stiffness of his right hand and arm, Butters could not return to his duty in the dock-yard until Monday, the 11th of October.

" *Remarks.*—Eight days nearly elapsed here, between the receipt of the local injury and the commencement of fever. Yet there can be no doubt that the latter was consequent on the former; or, in other words, that the injury was the cause, and the fever the effect.

"There can be no doubt, also, that this was the same sort of disease which prevailed amongst the mechanics in the dock-yard about the same time. Vesications were, however, wanting in the arm to characterise genuine erysipelas; but there was tumefaction, redness (disappearing on pressure), and fever. The disease called by Mr. Pearson *œdema cum erythemate* resembles this appearances, but the violence of fever constitutes the distinction.

"As Butters recovered, I made a point of seeing him at my own house on the 23d of January, 1825. He informed me that a tightness and smarting of these two fingers were still felt when he attempted to grasp any large body; owing, I suppose, to adhesions formed between the extensor tendon and its sheath." (P. 12—21.)

Having, in the above cases, given our readers some notion of the disease in question, we shall proceed to state some of the causes to which it has been ascribed; first observing, that our author devotes two or three pages to the consideration of whether the complaint was identically the same in all the individuals attacked; a question which he does not seem prepared to answer absolutely in the affirmative, although, with respect to six or seven of the patients, there can be but little doubt that such was the case. Dr. Butter mentions five distinct modifications of the dock-yard disease, which are as follows:—

"1. Fever with inflammation of the absorbent system.
"2. Fever with cellular inflammation and suppuration.
"3. Fever with erysipelas and vesications, or gangrene.
"4. Fever with erythematous patches.
"5. Fever with intense local pain, void of redness."

—and argues for the identity of the disease from analogies drawn from the differences to be found in measles, in scarlatina, in erysipelas, and even in the plague: nay, his last analogy is still stronger; it is the cases of Mr. Egan and Dr. Dease, who were infected with a kindred disease from dissecting the same body, and yet wide differences resulted in the symptoms. Our author concludes this part of his argument thus:—

"Fifteen men, in working health, receive slight wounds, either punctured, lacerated, contused, or abraded, too trifling to exempt them all from their duty. The majority pursue their employments, and in the course of time the wounds create fever and irritation, of which death was a common result.

"If this be not identity—perfect sameness, why did fever appear after the receipt of the mischief? How was it restrained prior to the local injury? Why should the disease generally arboresce around the provoked parts, and ramify through the cellular tissue, disturbing the nervous system, and carrying destruction in its growth?

"They made no complaint before the infliction of the wounds; they all suffered fever, which could be either directly or subsequently referred to their injuries; and those who died, with the exception of Henwood, did not survive the fifteenth day. Their febrile symptoms were sufficient to characterise the disease generally as one and the same genus; and it would be the height of scepticism any longer to doubt its identity. The fact is, there existed in each man a propensity to excitement, which was roused into action by any kind of external irritant, no matter what." (P. 131.)

With respect to the causes that have been assigned, we find that suspicion has fallen upon the teak-wood, the mineral tar, the state of the atmosphere, and even upon the dressings employed to the wounds. We need scarcely, however, remark, that these surmises are groundless, and are fully refuted, both by the nature of the accidents and the history of the cases.

We cannot pretend to follow our author through the remaining portions of this division of his work. He touches upon almost every subject that bears any relation to irritative fever, from whatever cause produced;—he shows an intimate acquaintance with all the modern writings connected with the subject, but he throws upon it no new light of his own. We therefore pass on to an enumeration of the leading symptoms of the disease, and the morbid appearances found on opening the bodies of those who died. Of the former, rigors, external redness, great tendency to gangrene, nausea, and deranged secretions of the stomach and bowels, great irritability, and excessive pain in the parts affected, formed the most prominent. The blood is described as having been firm, buffed, and cupped, in some instances, and free from inflammatory crust, and loose in its texture, in others. From the pulse our author draws no particular inference: the frequency, he says, was always increased after bleeding; but is not this generally the case?

Under the head *inflammation*, Dr. Butter offers some arguments tending to prove that real inflammation did not exist in this disease; and he, therefore, proposes to call it *irritative fever.*

With respect to the morbid anatomy, he has not much to say. Only four of the twelve bodies were examined after death. Gangrene on the surface is mentioned, with intolerable fœtor; the bodies running into rapid decomposition. In the abdomen of three of the men, there was a remarkable lividity about the junction of the ilium and cœcum, and a colouring of the peritoneum, illustrative of previous congestion. In the stomach of Bate, there was a reddish patch near the pyloric orifice. Around the kidneys of two, a collection of matter had formed. Finally, in the brain, we are told that evidence existed of the venous sinuses having been gorged with blood.

Relative to the prognosis in this disease, we need, surely, say no more than that, out of fifteen, only three persons recovered; though there does not seem to be any one symptom, in particular, which could be called a certain guide in forming a judgment as to the probable result: hiccough, wandering, and a sinking about the præcordia, are considered by our author as among the most formidable.

With respect to the mode of treating this disease, we can only refer our readers to the cases we have detailed; since we find it impossible to follow our author through his very extended disquisition as to the utility of each particular remedy in irritative fever. In this inquiry, he has not confined himself to the disease of the dock-yard, but has quoted the opinions of almost every writer of modern days, (and some, indeed, of the ancients,) from POTT and WILLAN to Dr. COLLES and DUNCAN. Even this is accompanied with not a few digressions respecting the various species of bark, and the sulphate of quinine,—the general advantages and disadvantages of blood-letting,—besides a review of the principal local remedies that have been recommended at various times, and by various authors. We must be, therefore, contented to give his summing-up of the evidence respecting the disease of which he more particularly treats, which we shall do in his own words.

"I believe, now, that I have enumerated a sufficient number of facts to direct the practitioner to that treatment which will be found most proper and successful in controlling irritative fever or erysipelas excited by local injuries, either of the simplest kind or aided by a poison. The consitutional treatment will be very much the same, although the local may differ. After the maturest deliberation, which I have given to the subject, I venture, with deference, to offer the following corollaries, hoping that they will be considered as the only explanations which I can advance, respecting the origin, causes, nature, and treatment, of this disease:—

"1st. That the constitutions of these men had been rendered unusually susceptible of irritation at this time.

"2dly. That diet, air, their occupations, and other circumstances influencing animal health, had collectively operated in creating this morbid propensity.

"3dly. That the addition of an external injury to a susceptible habit of body, formed the disease.

"4thly. That any kind of local excitement sufficed.

"5thly. That the local injuries would have proved inadequate to the causation of this disease, in persons not predisposed to excitement.

"6thly. That the disease was not confined to the artificers of the Royal Naval Yard at Devonport, as erroneously supposed; but was recognised, in detached instances, amongst other people in the neighbourhood.

"7thly. That the disease was the traumatic erysipelas of some, and the irritative inflammation of other authors.

"8thly. That its leading character was a tendency to spread, to form sloughs, or unhealthy fluids, and to leap from part to part.

"9thly. That bleeding, and other depletory measures, proved inadequate to the cure of this disease.

"10thly. That two cases of recovery arose, probably, from the restoration of healthy secretions; and the third, from the removal of tension by incision.

"11thly. Experience has shown that the object should be, to invite and keep the disease to the surface by local measures.

"12thly. That the general treatment ought to be calculated to invigorate and strengthen the constitution, with the view of expelling the aggressions of disease towards the interior." (P. 300—302.)

It will now be apparent to our readers, that our correspondent (Mr. Tripe) and Dr. Butter have formed opposite conclusions, both as to the nature and treatment of this disease; the former urging the necessity of prompt and vigorous blood-letting, the other drawing his inferences from the fatal termination of the majority of the cases, (in most of them blood having been freely drawn, upon the appearance of general derangement of the system,) and dwelling with considerable force upon the observations and cases which have lately appeared before the profession, in support of an opposite mode of treatment; the most forcible illustration of which, perhaps, is to be found in the relation of Dr. A. T. Thomson's case, and in Mr. Shaw's paper. Between these two conflicting opinions we may, perhaps, be allowed to interpose a middle term. It appears to us, from the relation of the cases in Dr. Butter's work, as well as from that of Quick, as detailed by Mr. Tripe, that both systems are right, and both are wrong, under certain circumstances. The case of Quick impresses upon us the notion of a man labouring under phrenitis in its most acute form, and we should have been inclined to have treated it entirely upon that view; whereas, in the history of Bate, of Dr. Bell, and some others, the practice adopted in Dr. Thomson's case, and the explanation of the phenomena as given by that gentleman, appear to us to be most reconcileable to the facts detailed. Upon the whole, we should be inclined to think that, where blood-letting and the depletory system is determined on, it should be employed as soon as re-action has become established,—and then used boldly; for it would seem, in many of the cases, that syncope, hiccough, and other signs of great and irretrievable exhaustion, were the consequences of blood-letting after the first days of the disease had gone by. Such, for instance, was the effect produced by bleeding in the cases of Cowle, Horne, and Taylor: the first, on the seventh day from the commencement of the fever; the two latter on the fourth day.

We conceive Dr. Bell's case so full of interest, that we shall take an opportunity of publishing it entire in a subsequent Number of this Journal.

---

### DIVISION II.

### FOREIGN.

ART. III.—*Tableau des Maladies observées à l'Hôtel Dieu, dans les Salles de Clinique de* M. le Professeur RECAMIER, *pendant le premier Trimestre de* 1825. Par L. M. MARTINET.*

*An Account of the Diseases observed at the Hôtel Dieu, in the Clinical Wards of* Mr. Professor RECAMIER, *during the first three Months of* 1825. By M. L. MARTINET.

WE have frequently had occasion to allude to the numerous and interesting pathological facts, which we have derived of late years from the zeal, the industry, and the opportunities of the French. We have sometimes, likewise, although less frequently and with less satisfaction, commented on their practice, principally with a view of pointing out wherein we might derive useful hints in our treatment of disease. Acknowledging, as we freely do, the obligations they have conferred on pathology, or, more properly, on morbid anatomy, we cannot help suspecting that one of the great causes of their superiority in this respect, is their inferiority in the only useful purpose of pathological knowledge—its application to the prevention and cure of disease. Anxious, however, to render unto Cæsar the things which be Cæsars, and willing to avoid the national prejudice, at which we smile, in the modern writers of France, Italy, and Germany,† we have preferred laying before our readers specimens of their opinions and practice, faithfully taken from their writings, rather than expressing general conclusions of our own. We have thus, as it were, left the doctrines of our neighbours to find the level of their merits without our assistance; and, on the present occasion, we mean not to depart from the method we have formerly pursued, but proceed to give an account of the diseases treated, and the treatment adopted therein, at the principal hospital in Paris, during the first three months of the present year.

The total number admitted during the above period was 187, of whom thirty-two died, being nearly one-sixth; there were

---

* *Revue Medicale*, Avril.

† We allude especially to BROUSSAIS, TOMMASINI, and AUTENRIETH. The opinions of the two former are probably familiar to our readers; those of the latter are to be found in a work entitled *Uebersicht über die Volks-Krankheiten in Gross-Brittanien, &c.* a very satisfactory review of which is contained in the *Edinburgh Medical and Surgical Journal*, April 1825.—REVIEWER.

131 men, and 56 women; 143 were affected with acute diseases, and 44 with chronic: of the former, twenty-one died, or one-fifth; of the latter, twelve, being more than one-fourth.

The most common diseases were inflammations of the alimentary canal and respiratory organs, which, as well as the number who died of each, will appear from the following table:—

| | *No.* | *Deaths.* |
|---|---|---|
| Fevers { Intermittent | 1 | |
| Fevers { Nervous | 2 | — 2 |
| Cerebral congestions | 3 | |
| Arachnitis | 2 | — 2 |
| Various cerebral diseases | 3 | |
| Amaurosis | 1 | |
| Epilepsy | 4 | |
| Thoracic contusion | 1 | |
| Pulmonary catarrh | 18 | |
| Bronchitis | 7 | — 3 |
| Pleuro-pneumonia | 20 | — 5 |
| Pleurisy | 10 | |
| Phthisis | 9 | — 6 |
| Pericarditis | 2 | — 2 |
| Hypertrophia of the heart, with dilatation of the ventricles | 5 | 3 |
| Angina Pectoris | 1 | |
| Catarrhal affections without fever | 3 | |
| Catarrhal fever | 35 | |
| Severe fevers (*fievres graves*) | 18 | — 5 |
| Saburral fever | 5 | |
| Chronic gastritis | 2 | |
| Enteritis | 2 | |
| Disease of the rectum | 1 | |
| Painter's Colic | 6 | — 1 |
| Tænia | 1 | |
| Hepatitis | 1 | |
| Amenorrhoea | 1 | |
| Schirrus of the Uterus | 1 | |
| Abdominal hemorrhage | 1 | — 1 |
| Peritonitis | 4 | — 2 |
| Erysipelas | 3 | |
| Anomalous eruption | 1 | |
| Dartres | 2 | |
| Rheumatism | 7 | |
| Exostosis of the cubital bone | 1 | |
| Struma | 1 | |
| Curvatures | 2 | |
| Total | 187 | — 32 |

The solitary case of *ague* presented no circumstance worthy of remark, except that it was readily cured by opium, given at the commencement of the hot stage.

The two cases entered as *nervous fever* are curious, and require more notice. One of these, a young man of eighteen, who had been ill for a fortnight before he was brought to the hospital, had symptoms of a moderate catarrhal affection, to which had been added, during the last few days, head-ache, tendency to vomit, yellowness of the tongue, bitter taste in the mouth, and wandering pains in the abdomen. He had an enema, containing a grain of tartarised antimony. On the following day, the pain of the belly had entirely ceased, as well as the head-ache; but some fever still remained, and the epigastrium having become tender to pressure, twenty leeches were applied. The treatment, up to this period, had consisted of gum-water, and emollient fomentations to the belly, when (10th of February,) the patient, who had begun to get up, was seized with a nervous attack *(attaque des nerfs)*, which was of short duration, and left no sensible lesion behind it. He had never previously had any similar attack. Next day, when seen in the morning, he complained of oppression and general uneasiness, of which, indeed, his countenance was sufficiently expressive. He had acute pain at the lower part of the chest, and his pulse was small and concentrated. Twelve grains of musk were prescribed; but the patient having got up a few minutes after the visit, to go to the close-stool, expired while sitting upon it.

On examination after death, no morbid appearances were found capable of accounting for the event. We may observe, however, that the heart was pale and bloodless; and, perhaps, the case might more properly be regarded as one of death from syncope, rather than fever.

The other case entered as nervous fever we shall give at full length, principally with the view of making our readers acquainted with the treatment adopted.

"Gaspard Nuchelot, aged nineteen years, a turner, of robust constitution, ill for some days. Leeches had been applied behind the ears, and to the epigastrium, to counteract the head-ache and the looseness which existed.

"On the 6th of February, we found him in the following state:—His face was but little flushed, and scarcely altered; notwithstanding which, his answers were usually slow, although correct; the conjunctiva not injected, the pupils equally dilated, and but little contractile; the locomotive power of the limbs free; the sensibility of the skin unimpaired; the tongue white and moist; the belly not at all tender to pressure; the heat of skin and frequency of pulse very moderate.—Eighteen leeches behind the ears.

"7th.—The symptoms above mentioned continue unabated; the general sensibility begins to be blunted; some delirium during the night; belly becoming distended. (Thirty leeches behind the ears, bleeding;) blood rich and buffy.—Tepid bath with affusions; julep with extract k. k.* and ether; fomentations to the belly; toast and water for drink.

"8th.—No improvement; face little altered, although the intellect

* We do not know the composition of this: it is, we presume, a preparation of bark.

is plunged in a very marked state of stupor. The patient replied with difficulty and very slowly, and holds intercourse with those around him. The looseness continues, and the fever is rather higher than yesterday. —Blisters to the legs; bath; same julep.

"9th.—Ideas incoherent; pupils still dilated, and little contractile; general sensibility very much diminished; tongue becoming dry; fever moderate.—Twenty-five leeches behind the ears. In other respects as before.

"Night.—Agitated and delirious.

"10th.—Same as yesterday, but the tongue more dry; no rigidity of the limbs; abundant diarrhœa.—Marshmallow; bath; fomentations to the belly.

"11th and 12th.—State of intellectual stupor sensibly diminishing; the patient more readily holds intercourse; the fever continues, but as moderate as heretofore; the appearance of the face better; the tongue still dry, and the looseness continues.—Same treatment.

"13th.—To the symptoms above described are now added trembling of the fingers, particularly of the left hand, with some degree of rigidity of the upper extremities, particularly the right; the sensibility of both sides diminished; the pulse frequent and small. At night, agitated.—Same treatment.

"14th.—Countenance more and more altered; the same symptoms continue.

"15th.—Died this morning.

"*Examination of the Body, twenty-six hours after death.—Head:* Pia mater and arachnoid transparent, without the slightest thickening; very slight injection of the portion of the pia mater which covers the upper part of the left hemisphere. The brain, cerebellum, and cerebral protuberance, in their natural state. No serum in the ventricles, at the base, or surface of the brain. Dura mater perfectly sound.—*Chest:* Lungs crepitating, light and healthy even at their posterior part; pleura, heart, and pericardium, in their natural state.—*Abdomen:* Mucous membrane of the stomach, duodenum, jejunum, ilium, and cœcum, not at all softened, and in the best possible state. Wrinkles of the stomach and duodenum projecting, and covered with tenacious bile; the descending colon having a slight, red, and very fine arborisation, extending about three fingers' breadth. The feculent matters perfectly figured. Liver, spleen, kidneys, and bladder sound."

This case is regarded as very extraordinary, in as much as no morbid appearances, which presented themselves after death, were sufficient to account for that event. It is true that the disciples of Broussais will find it rather a hard matter to demonstrate the existence of their favourite gastro-enteritis; nor will it be much easier for the followers of Dr. Clutterbuck to make out cerebral inflammation. But in this country, we believe, there are not many practitioners who are not quite aware that death frequently takes place in fever, without any lesions being left behind of such a nature as to be traced with the knife; and, generally speaking, we are more accustomed to regard them, when found, as the consequences, rather than the cause, of the disease.

*Diseases of the head.*—These consisted of three cases of cerebral congestion; of which one, occurring in a person of twenty, and having produced paralysis of the arm and tongue, immediately yielded to bleeding. An arachnitis, limited to the inferior surface of the cerebellum, with formation of false membranes on the left lobe of this organ, and on the same side of the tuber annulare, affording an opportunity of confirming the justice of M. RECAMIER's diagnosis. One circumstance in this case deserves attention: in the midst of the extreme disorder in which the patient was plunged, the pulling back of the head, the opisthotonos, and the strabismus of the left eye, &c. the intellectual faculties were preserved, and enabled the patient to complain of a violent head-ache and pain at the pit of the stomach, and to put out his tongue when desired, even till within a few moments of his death. This fact is in accordance with the observations of M. MARTINET,* with regard to the difference in the symptoms corresponding to the seat of the inflammation. According to this pathologist, delirium only occurs when the membrane covering the hemispheres is affected.

The other cases of arachnitis presented nothing remarkable; and only one of three entered under the head of "*various Cerebral Affections*" deserves attention. It consisted in a sudden and transient affection of the mouth, preceded by some confusion, loss of memory with regard to words, and an inability to articulate particular sounds. Thus, to express that he had no pain in the head, he said, "*les douleurs ordonnent un avantage;*" and, being required to write his answer, he immediately wrote "*je ne souffre pas de la tête,*" and so on in other instances; being generally able to give a distinct answer in writing, although he could not afterwards read it. We have ourselves known a patient, under similar circumstances, make use of one sound to express every thing, apparently without any idea of speaking differently from others, and expressing astonishment at not being understood. Peculiarities of this kind, we believe, are not very uncommon. The patient above alluded to by M. Martinet was discharged from the hospital without benefit; and the only treatment mentioned is "roasted coffee in doses of an ounce."

In *four epileptics*, M. Recamier tried the bark of the pomegranate, in doses of half an ounce; afterwards sugar of lead combined with oxide of zinc, assafœtida with extracts of chamomile, and hyoscyamus. In three women, colic and diarrhœa

* *Recherches sur l'Arachnitis*, p. 207, &c.

were produced; in one man, the remedies did neither good nor harm: he left the hospital too soon, however, to give them a fair trial. In the women, cupping-glasses were afterwards frequently applied along the vertebral column, but without any advantage.

*Diseases of the Chest.*—Pulmonary catarrh was both a frequent and obstinate disease. Bleeding, either general or local, in the first instance, and subsequently blistering, were found the best remedies. In this, the treatment differs nothing from that usually adopted among us. The Phellandrium aquaticum, in doses of from twelve to sixty grains, united to the syrup of bark, is stated to have diminished the violence of the cough and the quantity of the expectoration, in several of these cases, and in some of phthisis. Hydrocyanic acid, to the extent of four and five drops in three ounces of fluid, did not, in general, produce the quieting effect expected from it.

Frequent opportunities presented themselves of verifying the existence of a disease, which has not been described by those who have treated *ex professo* on the phlegmasiæ of the chest: we allude to inflammation of the minute ramifications of the bronchiæ. M. Andral is asserted to be the only one who has mentioned this affection, and by him it is noticed in a very cursory manner. In inflammation of these parts, the secretion is vitiated, and the chemical phenomena of respiration are incomplete: hence the disturbance in the functions of breathing and circulation. The dyspnœa is aggravated by fits, and then becomes extreme; the action of the heart acquiring a frequency proportioned to the acuteness of the attack. Finally, the patients are reduced to a state of true asphyxia, which speedily proves fatal; the inflammation of the air-vesicles rendering impossible the proper disoxygenation of the blood. On the subject of this imaginary discovery, M. Martinet promises speedily to produce a separate memoir. What information it may contain, we of course cannot presume to say; but we are well satisfied that there is nothing described in the paper before us which is not familiar to most practitioners in this country, and which has not been described by various well-known writers. It is true we do not pretend to be able to say, so far is the mucous membrane inflamed, and no further; but there are certain symptoms,—such as an oppression, with deep-seated but dull pains in the chest, rapid but not very hard pulse, with that state of respiration and appearance of countenance which accompanies the imperfect arterialisation of the blood,—by which we are accustomed to detect the presence of inflammation of the mucous membrane of the lungs; and, so long as our practice is measured by the urgency of the symptoms, it matters not a jot whether the inflammation be situated in the very extremities of the air-tubes, or half-an-inch to many half-inches nearer the larynx. Out of seven patients, four recovered,—one very speedily, three slowly and with difficulty. Active antiphlogistic means were adopted in all.

In the cases of *pneumonia* and *pleurisy*, similar treatment was adopted, and with more activity than we generally give our continèntal neighbours credit for pursuing. Bleedings from the arm were frequently repeated, and cupping glasses applied over the seat of the pain: when in this manner the urgency of the symptoms had been subdued, blisters were applied to the side; and we are told that, "in many instances, blisters to the legs restored the patients from a state of sinking, which excited fears of a fatal result." The only other circumstance deserving remark, is the presence, throughout the whole course of the disease, in one case, of violent hiccough, which resisted all antispasmodic means, and afterwards spontaneously disappeared during convalescence.

It is remarkable that, while no case of *pericarditis* occurred at the Hôtel Dieu during the year 1824, no fewer than three examples should have been met with during the month of March, 1825: of these the only fact worthy of observation is, that in one of them the existence of the disease was not marked by any symptom which led to its detection during life.

*Diseases of the Abdomen.*—"Catarrhal fevers were the predominant affections." Some of these were slight, only requiring diluent drinks; but the greater number were treated with the repeated application of leeches to the different parts of the abdomen which were the seat of pain, and a few required general bleeding. In eighteen the symptoms were severe, consisting of considerable tendency to sinking, sordes of the mouth, and dryness of the tongue. In such cases, tepid baths and affusions were employed, in addition to the measures above mentioned, particularly when the fever and stupor were considerable: they were generally attended with success. In some instances, M. Recamier ordered blisters to be applied to the legs; "and frequently we perceived the disease became less severe as a consequence of their application."

The *catarrhal fever*, as it is here called, readily yielded to the antiphlogistic treatment, when it was free from saburral complication. Where, however, this did exist, evacuant remedies immediately carried off the disease, while the antiphlogistic plan did no good. "In the saburral fever, the secretion of mucus becomes vitiated, the fluid has changed its nature; the inner membrane of the stomach is smeared over with a tenacious mucus. Emetics succeed admirably in modifying the gastric secretion, and in augmenting the quantity secreted, which then clears the surface of the stomach; as we perceive the tongue become moist and clean after the nausea resulting from an emetic. Such are the characters which M. Recamier assigns to the saburral fever, which, as we see, differs entirely from the catarrhal; this last being altogether of an inflammatory nature, and requiring, in consequence, an antiphlogistic treatment." In four individuals eschars formed, without having been preceded by any signs of inflammation: these were regarded as crises, the disease ceasing when they made their appearance.

Of six individuals affected with *painter's colic*, three were successfully treated with leeches. In one of these the pain ceased, "*comme par enchantement*," on the application of fifty leeches; no diminution of pain having accrued from the treatment adopted at La Charité. This method was adopted even in those cases in which the disease appeared least of an inflammatory character. In the other three, the narcotico-purgative system was found to answer best. One of these patients, after having been discharged, was obliged to return to the hospital for the same complaint, and was killed by the acupuncturation: his case was detailed in our Number for June.

The only other case is one in which a tumor filled with blood was found in the abdomen of a young man; and this case likewise we have previously given in the Number above alluded to.

---

